# A school-based intervention to reduce overweight and inactivity in children aged 6–12 years: study design of a randomized controlled trial

**DOI:** 10.1186/1471-2458-8-257

**Published:** 2008-07-25

**Authors:** Wilma Jansen, Hein Raat, Evelien Joosten-van Zwanenburg, Ivo Reuvers, Ron van Walsem, Johannes Brug

**Affiliations:** 1 Department of Public Health, Erasmus MC, Rotterdam, The Netherlands; 2 Rotterdam Public Health Service and Environs, Rotterdam, The Netherlands; 3 Municipal Sport and Recreation Department, Rotterdam, The Netherlands; 4 EMGO Institute, VU University Medical Centre, Amsterdam, The Netherlands

## Abstract

**Background:**

Effective interventions to prevent overweight and obesity in children are urgently needed especially in inner-city neighbourhoods where prevalence of overweight and inactivity among primary school children is high. A school based intervention was developed aiming at the reduction of overweight and inactivity in these children by addressing both behavioural and environmental determinants.

**Methods/design:**

The main components of the intervention (Lekker Fit!) are the re-establishment of a professional physical education teacher; three (instead of two) PE classes per week; additional sport and play activities outside school hours; fitness testing; classroom education on healthy nutrition, active living and healthy lifestyle choices; and the involvement of parents. The effectiveness of the intervention is evaluated through a cluster randomized controlled trial in 20 primary schools among grades 3 through 8 (6–12 year olds). Primary outcome measures are BMI, waist circumference and fitness. Secondary outcome measures are assessed in a subgroup of grade 6–8 pupils (9–12 year olds) through classroom questionnaires and constitute of nutrition and physical activity behaviours and behavioural determinants. Multilevel regression analyses are used to study differences in outcomes between children in the intervention schools and in control schools, taking clustering of children within schools into account.

**Discussion:**

Hypotheses are that the intervention results in a lower prevalence of children being overweight and an improved mean fitness score, in comparison with a control group where the intervention is not implemented. The results of our study will contribute to the discussion on the role of physical education and physical activity in the school curriculum.

**Trial registration:**

[ISRCTN84383524]

## Background

Effective interventions to prevent overweight and obesity in children are urgently needed.[1] The prevalence of childhood overweight and obesity is increasing worldwide with all its consequences for immediate health, already apparent from increasing health care costs for obesity related morbidity in youth, as well as for health in later life, due to tracking of overweight and obesity into adulthood. [2-9]

The increase in childhood overweight and obesity can be attributed to behavioural and social ecological factors causing long-term imbalance between energy intake and energy expenditure. [10,11] In fact, the environment has been recognized more and more as 'obesogenic' agent in the aetiology of obesity. [12-15] Physical, socio-cultural, economic and political environmental influences on energy balance related behaviours can be distinguished at the micro level (households, schools, neighbourhoods) as well as at the macro level (health care, media, public transport, town planning).[14] Programmes on the prevention of childhood obesity should therefore address both behavioural and environmental determinants.

Many obesity prevention programmes have been developed and evaluated, but so far only yielded 'best practice' recommendations. A recent, large synthesis research of 147 programmes on prevention and treatment of childhood obesity over the last two decades revealed that engagement in physical activity (PA) is a critical intervention in childhood obesity prevention programmes.[1] These findings are supported by other reviews. [16-20]

The school emerged as a critical setting [1]. In a review of 25 school-based childhood overweight prevention programmes 17 of 25 were effective based on a statistically significant reduction in body mass index or skin-folds in the intervention group compared to the control group.[21] Another review included 14 intervention studies in the school arena, of which half were successful and had an effect on either overweight or obesity.[22]

In the Netherlands most recent figures demonstrate that prevalence rates of overweight in 4–16 year olds are rising at an even faster rate than before. Prevalence rates of overweight (including obesity) reached 14.5% for boys and 17.5% for girls in 2003 as compared to 9.7% and 13.0% in 1997 and 3.9% and 6.9% in 1980.[23] The largest increase in prevalence of overweight and obesity in the Netherlands occurred among primary schoolchildren [24] and the highest rates of childhood overweight and obesity are found in ethnic minorities and metropolitan areas [25].

Figures on the amount and trends of PA in Dutch primary school children are largely lacking, but a recent study on physical activity in relation to the physical environment in the Netherlands demonstrated that only 3–5% of the primary schoolchildren in inner-city neighbourhoods was physically active for the recommended one hour a day, as measured by self-report and accelerometry.[26]

Apparently, relatively high prevalence rates of childhood overweight and obesity coincide with low rates of PA in inner-city neighbourhoods, at least in The Netherlands, urging schools and local governments to take action.

In order to contribute to the prevention of overweight in primary schoolchildren, a school based intervention was developed targeted at the reduction of overweight and inactivity in primary schoolchildren attending schools in inner-city areas in Rotterdam addressing both behavioural and environmental determinants. This paper describes the intervention and the study design for assessing the effectiveness of the intervention.

## Methods/design

### The intervention

The intervention Lekker Fit!, which can be translated as 'enjoy being fit', focuses on the promotion of healthy eating behaviour and active living rather than the achievement of an ideal body weight. By choosing this focus the intervention aims to reduce the chance of stigmatization of overweight children and of contributing to eating disorders or distorted perceptions of body image. [1,27,28]

The intervention targets individual behaviours as well as the environment and is based on the theory of planned behaviour [29,30] and the ecological model of Egger and Swinburn.[31] According to the theory of planned behaviour a given behaviour can be predicted form the intention to display that behaviour. The intention in turn is predicted by attitude, social influence and self-efficacy. The model acknowledges perceived behavioural control as a potential barrier between intention and behaviour. Within the ecological model the intervention concentrates on the physical and socio-cultural environmental influences on energy balance related behaviours, especially PA, within the micro environment of schools and to a lesser extent of home environments.

The targeted population consists of children attending primary schools in the more deprived, inner-city areas of Rotterdam where prevalence rates of overweight and obesity are relatively high.[32]

The intervention consists of multiple components, which will be described below.

### Intervention components

#### Three physical education (PE) classes a week by a professional PE teacher

The first component of the intervention constitutes a structural change in the school environment by the implementation of three PE sessions a week during school hours by a professional PE teacher for grades 3 through 8 (6–12 years of age). The usual curriculum of primary schools consists of two PE sessions a week by the classroom teacher or a professional PE teacher, dependent on the schools policy. The PE teachers of the intervention are paid for and supervised by the Municipal Sport Department for two years and arrange their lessons according to a standardised protocol.[33]. Participating schools express the intention to keep the PE teacher after the two year intervention period.

#### Sport and play activities outside school hours

A second component of the intervention is the organisation of additional sport and play activities outside school hours. These non-curricular activities are organised by the PE teacher and can be attended by the children on a voluntary basis. The focus of the activities is enjoying physical activity. Fun activities like rope skipping and dance are examples of the organized activities.

The total number of days a week children can be involved in sport and play activities inside and outside school hours ranges between 3 (only within school hours) to 5 (within and outside school hours).

#### Cooperation with Sport clubs

A third component of the intervention is the cooperation with local sport clubs and professional sport clubs. Local sports clubs are given the opportunity to present themselves during PE classes and outside school hours in order to let pupils get acquainted with several types of sports and promote sport club membership. Moreover, sport clubs are encouraged and supported by the Municipal Sport Department to establish satellite clubs in the more deprived neighbourhoods.

#### Eurofit test and Fitmeter

At the beginning and at the end of the school year the Eurofit test is administered by trained staff from the Municipal Sport Department during PE class.

The Eurofit test comprises of measurements of height, weight and nine different fitness tests, i.e. measuring balance, endurance, flexibility, power, speed and strength as shown in table 1.[34] The skin fold measurements that originally are part of the Eurofit test battery are replaced by the simpler and quicker measurement of waist circumference.

**Table 1:** Description of the Eurofit test components, dimensions tested and units of measurement.

Test component	Dimension	Description	Unit of measurement
Flamingo balance	Balance	Standing for 1 minute on one leg, while holding the other leg bend backwards in one hand	Number of attempts that were needed
Plate tapping	Speed	Tapping 2 plates (edges 60 cm apart) alternately, with the preferred hand, until each plate was touched 25 times.	Time needed measured to the nearest 0.1 second.
Sit and reach	Flexibility	Bending the trunk and reaching forward as far as possible while sitting on the floor with stretched legs and with the feet placed against a test box with a ruler placed on the top of the box.	Difference between feet soles and the tip of the largest finger measured in cm.
Standing broad jump	Power	Jumping from standing position	Distance in cm
Hand-grip	Strength	Squeezing a hand-dynamo meter as hard as possible with preferred hand	Kg to nearest 0.5 kg
Sit-ups	Endurance	Making as many sit ups as possible for 30 sec	Number of sit ups
Bent-arm hang	Endurance	Maintaining a bent arm position with an over-grip as long as possible while hanging from a bar.	Duration measured to the nearest 0.1 sec.
10 × 5 m shuttle run	Speed agility	Running as fast as possible 10 times between 2 lines, 5 m apart.	Time in sec.
20 m shuttle run	Endurance	Running 20 m forth and back with an initial running pace of 8.0 km/h and a progressive 0.5 km/min raise of the running speed given by a sound.	Last completed stage with a precision of 0.5.

**Figure 1:**
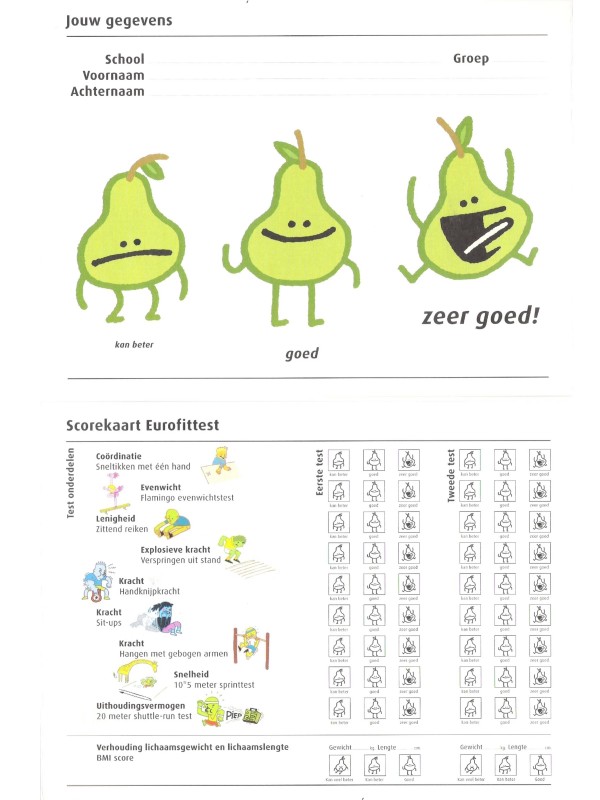
Score card with individual fitness scores, height, weight and weight status.

Children receive a score card (see figure 1) to take home with their test results compared with reference scores [35]. When their BMI is above age and gender specific thresholds for overweight [36] parents receive a letter and are offered individual counselling by the school nurse. When needed motoric remedial teaching is offered.

All individual Eurofit scores are stored in a web-based computer application – the Fitmeter – that was especially developed for this purpose and allows PE teachers to follow the development and progress of individual pupils and classes in comparison with reference scores. Additionally, the Fitmeter offers PE teachers a planning module for within and after school hour's activities and a registration module for attendance to voluntary activities of individual pupils.

Parents provide informed consent for storing test results in the Fitmeter and sharing individual scores with the Municipal Sport and Recreation department and Municipal Health Department for evaluation purposes.

#### Classroom education

Three classroom lessons and an introduction lesson are developed for all grades. The three lessons deal with healthy nutrition, active living and healthy lifestyle choices and are provided by the regular classroom teacher, who receives an extensive manual on the lessons. Central theme of the lessons is to enjoy a fit and healthy lifestyle. Each lesson starts with a homework assignment to be completed with the help of the parents. Assessment and awareness of the child's behaviour are the central themes of the home assignment. Each classroom lesson consists of a theoretical and practical part, during which knowledge is transferred and subsequently applied through activities like games, puzzles and tests. Each lesson finishes with goal setting by drawing up a joint agreement regarding lifestyle for the period until the next lesson. Education material and classroom posters for writing down the agreements are part of the provided material .

#### Parent involvement

Parents are important agents in shaping children's eating and physical activity behaviours. [27,37-40] Besides the homework assignments and fitness score card, parents are involved by providing them with written information on the intervention and inviting them for a gathering at the beginning of the school year. During this gathering information is provided by the school nurse or a dietician about a healthy lifestyle, focusing on reducing sedentary activities (watching TV and playing on the computer), promotion of outdoor play, and reduction of sugar-sweetened beverage intake and promotion of having breakfast daily. All of these behaviours have been shown to be associated with childhood obesity.[41,42]

### The study on the effectiveness

#### Study design and procedures

A cluster randomized controlled study design is used to evaluate the effectiveness of the intervention with baseline measurements at the beginning of the school year 2006/2007 and follow-up measurements at the end of the same school year.

The main outcome measures consist of BMI, waist circumference and fitness and are measured among all pupils of grade 3 through 8 by trained staff during physical education class. High levels of habitual PA and increases in PA have been shown to be associated with improvements of fitness in children [43-46].

Secondary outcome measures consist of selected energy balance related behaviours and possible mediators and moderators as described in the Environmental Research Framework for weight Gain prevention.[12] In this framework energy balance related behaviours are influenced by environmental influences in a direct way or an indirect way through cognitive mediators (attitude, subjective norm, perceived behavioural control and intention).

Moderators of the relations between environmental influences and cognitive mediators on the one hand and energy balance related behaviours (i.e. specific nutrition and physical activity behaviours that contribute significantly to energy balance) on the other hand, are personality, awareness, habit strength, clustering of behaviours and personal characteristics like gender, ethnicity, and socio-economic status. Secondary outcome measures are gathered via classroom questionnaires in a subgroup of the study population consisting of grades 6 through 8 (9–12 years of age), administered on a normal weekday except for Mondays and guided by the classroom teacher. The questionnaire was developed in the ENDORSE-study and adjusted to make it better applicable to primary schoolchildren.[47]

Parents receive written information on the study and provide their informed consent. Children in grades 6 through 8 also receive written information on the study. The study is approved by the Medical Ethics Committee of Erasmus MC.

#### Recruitment and randomization procedure

Primary schools in Rotterdam with large populations of foreign ethnicity were free to apply for participation in the intervention, which was already implemented on 30 schools in the school year 2005/2006. Spontaneous applications made further active recruitment unnecessary. Schools that applied for participation were informed of the study and were offered a chance of 50% to participate in school year 2006/2007 versus a chance of 50% to be allocated to the control group in school year 2006/2007 continuing with their usual curriculum and to participate in the intervention in the next school year 2007/2008. All of the 27 schools that spontaneously applied, agreed to participate in the study. Schools were paired according to size, ethnicity and neighbourhood into 13 comparable pairs. One school could not be paired and was excluded from the study. Randomization took place within each pair with the toss of a coin.

After randomization 3 pairs were lost to the study, due to withdrawal of schools (1 pair) and implementation of the intervention components prior to the study (2 pairs).

Eventually, twenty primary schools located in multi-ethnic, mostly low-income, inner-city neighbourhoods in Rotterdam participated in the study.

#### Measures

##### Anthropometric measures

Body mass index (BMI) was calculated using weight (kg) divided by squared height (in m). Height was measured to the nearest 0.1 cm using a commercial mobile stadiometer, and weight was measured to the nearest 0.2 kg using a flat electronic weighing scale (SECA 888) in light (sport) clothing following a standardized protocol [48]. Pupils were categorised as underweight, normal weight, overweight, or obese. Overweight and obesity were defined using the age and gender specific cut-offs that correspond to adult cut-offs for BMI of 25 and 30 kg/m^2 ^as published by the International Obesity Task Force (IOTF) [36]. Underweight was defined using the age and gender specific cut-offs corresponding to adult cut-offs for BMI of 18.5 kg/m^2 ^derived from the 1996/1997 Dutch national growth survey [49].

Waist circumference was measured over the naked skin using flexible bands (SECA) with an accuracy of 0.1 cm, half-way between the lower rib and the top of the iliac crest at the end of a gentle expiration. [50]

##### Fitness

Fitness was assessed by the Eurofit test. [34,35] See figure 1. The first 8 test components were administered during one PE session, the shuttle run test was administered one week later.

#### Questionnaires

##### Energy balance related behaviours

Physical activity was measured using the following questions: i) 'How long did you play outdoors after school yesterday?' ('did not'/'less than half an hour'/'1/2-1 hour'/'1–2 hours', '2–3 hours', 'more than 3 hours'); ii) 'On how many days did you do sport outside school hours last week?' (0 through 7); iii) 'How did you come to school today?' ('walking'/'cycling'/'public transport or car'/'other'); iv) on how many days last week did you walk or cycle to school? (0 through 5). Dutch norms on minimal requirements for physical activity for children state that children should be active at least 1 hour a day and engage in sports twice a week.[51]

Sedentary behaviour was assessed using two questions: i) 'How long did you watch television, DVD or video outside school hours yesterday?' ('did not'/'less than half an hour'/'1/2-1 hour'/'1–2 hours', '2–3 hours', 'more than 3 hours')'; ii) 'How long did you spend on the computer or game-computer outside school hours yesterday?' ('did not'/'less than half an hour'/'1/2-1 hour'/'1–2 hours', '2–3 hours', 'more than 3 hours'). A maximum of 2 hours screen time per day is the recommendation given by youth health care in the Netherlands [52].

Consumption of sugar-containing drinks was assessed with two questions, after giving examples of such drinks (i.e. lemonade, soft drinks, sport drinks, chocolate milk, yoghurt drinks, fruit juices; exceptions: light drinks, orange or grapefruit juice): i) 'How many glasses of sugar-containing drinks did you have yesterday?' (0 through 5 or more); ii) 'How many days last week did you take sugar-containing drinks to school?'(0 through 5). A maximum of 2 sugar-containing drinks a day is recommended by Dutch Youth Health care.[52]

Consumption of fruit (including orange juice and grapefruit juice), was assessed with two questions, after giving examples of pieces of fruit: i) 'How many pieces of fruit did you have yesterday?' (0, 1/2, 1, 11/2, 2 or more); ii) 'How many glasses of orange or grapefruit juice did you have yesterday?'(0, 1, 2 or more). Two pieces of fruit a day or one piece of fruit in combination with one glass of orange or grapefruit juice is the Dutch recommendation for children (9–12 years).[53]

Skipping breakfast was measured with two questions: i) Did you have breakfast this morning?' ('yes'/'no'); ii) 'on how many days last week did you have breakfast before going to school?'

##### Potential mediators and moderators

As potential mediators attitudes and intentions towards energy balance related behaviours were included in the questionnaire. Attitudes towards outdoor playing, doing sports, watching television, using computer, having sugar-containing drinks, having fruits and having breakfast were measured with two questions: i.e. "I think (selected energy balance behaviour) is ...."('very good'/'good'/'not good, not bad'/'bad'/'very bad') and "I think (selected energy balance behaviour) is ...."('very nice'/'nice'/'not that nice'/'not nice at all'), representing cognitive and affective attitudes. Intentions to engage in outdoor playing, doing sports, having fruits, having breakfast, and to reduce television time, computer time and consumption of sugar-containing drinks were measured with single-item questions: i.e. "Do you intend to increase your outdoor play, to (continue) doing sports, to reduce TV time, to reduce computer time, to reduce consumption of sugar-containing drinks, to increase consumption of fruits, to (continue) having breakfast in the coming 6 months?"('yes certainly'/'yes probably'/'maybe yes, maybe no'/'probably not'/'certainly not'.

As potential moderators perceived health ('very good'/'good'/'moderate'/'not well'/'bad'), body weight perception ('far too thin'/'little too thin'/'not thin, not fat'/'little too fat'/'far too fat'), weight worries ('no'/'a little'/'a lot'), being member of a sports club ('yes'/'no'), owning a bicycle, having a television in one's bedroom, having a computer and/or game-computer at home and questions on sport participation of father and mother ('never'/'seldom'/'once a week'/'more than once a week') and body size of father and mother using Stunkard's body figure rating scales [54] were included as single item questions in the questionnaire.

##### Socio-demographic characteristics

Socio-demographic characteristics included gender, age, ethnicity and postal code.

Ethnicity is determined by country of birth of mother and father according to definitions of Statistics Netherlands. If both parents have been born in the Netherlands, the child's ethnicity is defined as Dutch; if one or both parents are born in another country, ethnicity is defined according to that country; if both parents have been born in different foreign countries, the country of birth of the mother is deemed most important.

Postal code is used to determine neighbourhood income level. Most recent data on average 2003 personal gross income level per postal code are provided by Statistics Netherlands. Postal codes refer to on average 20 (SD 17) houses and 46 (SD 38) inhabitants.

##### Process measures

Process measures are taken from the Fitmeter, registration forms for classroom teachers and registration forms for the school nurse or dietician.

##### Power considerations and data analysis

Power calculations showed a total number of children of 2,778 children in 20 schools are needed to detect a difference of 0.45 kg/m^2 ^between intervention and control group, assuming a standard deviation of 3.0 kg/m^2 ^for BMI and an intraclass correlation of 0.01 [55], with a power of 0.80 en alpha 0.05 (one-sided) and accounting for 10% loss to follow up.

Multilevel regression analysis will be used to test for post-test group differences on the main outcome measures corrected for pre-test measurements. [56]

## Discussion

The intervention combines structural changes in the amount of PA children receive with behavioural change through the school curriculum. A specific element is the implementation of three PE classes a week by a professional PE teacher, while two PE classes a week by a classroom teacher constitute the usual mandatory curriculum. Another specific characteristic of the intervention is the targeting of a population with relatively low SES and a high proportion of migrant children. A population that has been underserved so far. [1]

Several evaluated obesity prevention programmes have targeted PE or increased PA in the primary school setting. [57-68] Most of these interventions altered the content of existing PE lessons [59-61,63-65,68], others increased PA in the classroom [62] or during breaks [66]. Only a few actually augmented the amount of PE lessons a week for six months [57] or for 8 weeks[67].

Strengths of the study are the use of a cluster randomized controlled study design, the size of the study and the objectively measured primary outcome measures of weight status and fitness.

Weaknesses of the study are that secondary outcome measures are derived from self-report questionnaires and no objective measure of PA is used. Furthermore, in the self-report questionnaires the choice was made to measure a large amount of concepts to cover all aspects of the intervention. To keep the length of the questionnaires acceptable for children in the age of 9–12 years many single item questions were used at the cost of using validated questionnaires. The debate on self-report by children largely concerns recall problems of actual energy balance related behaviours.[69] We took those into account by making recall periods short. Self-reports by children on determinants of energy balance related behaviours like attitudes and intentions have not been subject to debate.

We hypothesize that the intervention will reduce the number of overweight children and improve fitness scores due to increased physical activity in comparison with the control condition. Furthermore, we hypothesize that the intervention will impact positively on energy balance related behaviours and its determinants in the intervention group as compared to the controls.

The results of our study are especially important for decisions on the amount of PE classes in the usual school curriculum and the position of a professional PE teacher within this curriculum.

## Competing interests

The authors declare that they have no competing interests.

## Authors' contributions

WJ designed the study and drafted the manuscript. EJvZ participated in the design of the study. IR and RvW participated in the design of the intervention and provided feedback on the drafts. JB and HR provided critical feedback on the study and drafts. All authors approved the final manuscript.

## Pre-publication history

The pre-publication history for this paper can be accessed here:


